# Alleviation of Hyperglycemia Induced Vascular Endothelial Injury by Exenatide Might Be Related to the Reduction of Nitrooxidative Stress

**DOI:** 10.1155/2013/843657

**Published:** 2013-11-26

**Authors:** Qian Zhao, Chun-ling Xu, Hai-yan Xiong, Wen Huang, Mei Zhang, Yun Wang, Si-yu Wang, Wen Wang

**Affiliations:** ^1^Department of Physiology and Pathophysiology, School of Basic Medical Sciences, Capital Medical University, No. 10 Xitoutiao, You An Men Wai, Fengtai District, Beijing 100069, China; ^2^Nursing Department, Peking University Shougang Hospital, No. 9 Jinyuanzhuang Street, Shijingshan District, Beijing 100144, China; ^3^Department of Neurology, Beijing Friendship Hospital, Capital Medical University, No. 95 Yongan Street, Xicheng District, Beijing 100050, China; ^4^Department of Nephrology, Beijing Tongren Hospital, Capital Medical University, No. 1 Dongjiaomenxiang, Dongcheng District, Beijing 100730, China; ^5^Department of Geriatrics, Beijing Tongren Hospital, Capital Medical University, No. 1 Dongjiaomenxiang, Dongcheng District, Beijing 100730, China

## Abstract

We will investigate the effects of exenatide on vascular endothelial injury and nitrooxidative stress in hyperglycemia both in vivo and in vitro and explore the role of nitrooxidative stress in endothelium-protective action of exenatide. Healthy male Wistar rats were randomly divided into 4 groups: control, diabetes mellitus (DM) model, low dose of exenatide treatment, and high dose of exenatide treatment. In vitro study showed that, compared with control group, the DM rats exhibited a lowered endothelium-dependent relaxation and damaged structural integrity of thoracic aortas, and there was a significant increase in plasma nitrotyrosine concentration. These parameters were improved after treatment with either low dose or high dose of exenatide for 45 days. In vitro study showed that exendin-4 (the active ingredient of exenatide) attenuated HUVECs injury induced by high glucose, with improving cell viability and attenuating cell apoptosis. Exendin-4 also significantly alleviated the increased malondialdehyde (MDA), nitrotyrosine content, and inducible nitric oxide synthase (iNOS) expression induced by high glucose in HUVECs. In conclusion, this study demonstrates that exenatide treatment can alleviate the vascular endothelial injury, as well as attenuating the nitrooxidative stress in hyperglycemia, implying that the endothelium-protective effect of exenatide might be related to the reduction of nitrooxidative stress.

## 1. Introduction

Cardiovascular disease is a major cause of morbidity and mortality in patients with diabetes mellitus (DM). Hyperglycemic episodes are closely associated with the development of cardiovascular disease. Endothelial dysfunction, as an early pivotal event in cardiovascular disease, is strongly associated with the pathogenesis of DM [1, 2].

Hyperglycemia can increase the production of superoxide anion (^∙^O_2_^−^). Although (^∙^O_2_^−^) itself is chemically inert, when it combines with nitric oxide (NO) at a diffusion-limited rate, it becomes a highly reactive species, peroxynitrite (ONOO^−^). ONOO^−^ can initiate both nitrative and oxidative reactions with proteins, lipids, and DNA. A characteristic reaction of ONOO^−^ is the nitration of protein-bound tyrosine residues to produce nitrotyrosine, and the production of nitrotyrosine has been used extensively as a footprint for ONOO^−^ in vivo. Recent evidence has indicated that the vascular endothelial injury induced by hyperglycemia is associated with the enhanced nitrooxidative stress both in vivo and in vitro [3–6].

Glucagon-like peptide-1 (GLP-1) is an important endogenous incretin hormone, which stimulates glucose-dependent insulin secretion from the pancreatic islet cells and supports glucose homeostasis [7], as well as stimulating *β*-cell proliferation and inhibiting *β*-cell apoptosis, increasing insulin sensitivity, and inhibiting glucagon secretion and gastrointestinal motility [7–10]. In addition, it has also shown beneficial effects on both cardiac [11–13] and vascular endothelial functions [14–19]. However, native GLP-1 is degraded rapidly by the ubiquitous enzyme dipeptidyl peptidase-4 (DPP-4), resulting in a half-life of no more than 1~2 minutes [10], which limits its therapeutic usefulness in clinic. Recently more stable GLP-1 analogues, such as exenatide, have been approved for clinical use against type 2 diabetes mellitus (T2DM). Exenatide is a synthetic version of exendin-4, a 39 amino acid peptide originally isolated from the venom of the Gila monster. Exenatide shows 53% sequence homology with native GLP-1, while still binding effectively to GLP-1 receptor on pancreatic *β* cells for its insulinotropic effects. It is resistant to DPP-4, resulting in a longer circulating half-life time, about 2.4 hours. In addition to coordinated effects on glucose metabolism [20, 21], exenatide exerts endothelium-protective action similar to GLP-1 [22, 23]. However, until now, the underlying mechanisms are not fully understood.

There is evidence that the endothelial dysfunction induced by hyperglycemia is associated with the enhanced nitrooxidative stress. However, whether the endothelium-protective action of exenatide is related to reduction of nitrooxidative stress still needs much exploration to clarify. Therefore, in the present study, we will investigate the effects of exenatide on vascular endothelial injury and nitrooxidative stress in hyperglycemia models both in vivo and in vitro and explore the role of nitrooxidative stress in endothelium-protective action of exenatide.

## 2. Materials and Methods

### 2.1. Reagents

Exenatide was provided by Eli Lilly and Company (Indiana, USA). Exendin-4 (the active ingredient of exenatide) was provided by AnaSpec (CA, USA). Streptozotocin, acetylcholine (ACh), type I collagenase, albumin from bovine serum (BSA), and 3-(4,5-dimethylthiazol-2-yl)-2,5-diphenyltetrazolium bromide (MTT) were provided by Sigma-Aldrich (St. Louis, MO, USA). Noradrenaline bitartrate injection (NE) was provided by Hefeng Pharmaceutical (Shanghai, China). Iodine [^125^I]-insulin radioimmunoassay kit was provided by Puerweiye (Beijing, China). Nitrotyrosine ELISA kit was provided by Hycult Biotech (Uden, The Netherlands). In situ cell death detection kit, POD, was provided by Roche (Lewes, UK). Ac-DEVD-AFC (caspase-3 activity assay substrate) was provided by Enzo Life Sciences (Farmingdale, NY, USA). The malondialdehyde (MDA) chemiluminescence assay kit was provided by Nanjing Jiancheng Bioengineering Institute (Nanjing, China). Rabbit anti-*β*-actin monoclonal antibody was provided by Epitomics (Burlingame, CA, USA). Rabbit anti-NOS3 (C-20) polyclonal antibody, mouse anti-NOS2 (C-11) monoclonal antibody, and goat anti-VE-cadherin (C-19) polyclonal antibody were provided by Santa Cruz Biotechnology (Santa Cruz, CA, USA). Rabbit anti-von Willebrand Factor (vWF) polyclonal antibody was provided by Zhongshan Golden Bridge Co. (Beijing, China). SuperSignal West Pico Chemiluminescent Substrate kit was provided by Thermo Scientific (Rockford, IL, USA). Other chemicals and reagents were analytical grade.

### 2.2. Animals

The investigation conformed to the guidelines of the Institutional Authority for Laboratory Animal Care of Capital Medical University and had been specifically approved by this committee. The investigation complied with the Guide for the Care and Use of Laboratory Animals published by the US National Institutes of Health (NIH Publication Number 85-23, revised 1996). All surgery was performed under urethane anesthesia, and all efforts were made to minimize suffering.

34 healthy male Wistar rats weighing 150–180 g were provided by the Institute of Laboratory Animal Science, Chinese Academy of Medical Sciences (Beijing, China), and housed for at least 1 week before experimentation. Rats were randomly divided into 4 groups: control group (CON), diabetes model group (DM), low dose of exenatide treatment group (*E*_min⁡_), and high dose of exenatide treatment group (*E*_max⁡_). Rats in the last three groups (*n* = 9/group) were fed with high fat emulsion (lard (20%), propylthiouracil (1%), cholesterol (5%), sucrose (5%), fructose (5%), sodium glutamate (1%), and salt (6%), emulsified in 20% Tween 80 and 30% propylene glycol) for 14 days first. Then oral glucose tolerance and fasting insulin level were detected to show whether insulin resistance had developed or not. For rats which have developed insulin resistance, streptozotocin (4 × 10^−2^ mg/g/d) was injected intraperitoneally twice to destruct the pancreatic *β* cells as previously reported [24]. Fasting blood glucose (FG) levels were measured 72 hours after the last streptozotocin administration to show whether diabetic symptoms had developed or not. Rats with FG levels higher than 11.8 mmol/L were judged as successful DM models. DM models rats were treated with exenatide, low dose (156.25 × 10^−3^ *μ*g, bid, injected hypodermically), or high dose (312.5 × 10^−3^ *μ*g, bid, injected hypodermically) for 45 days. All rats were fed a standard rodent diet and provided with water freely.

### 2.3. Oral Glucose Tolerance Test (OGTT) and Fasting Insulin (FIns) Detection

Blood samples were collected from the tail veins in conscious rats to measure blood glucose and plasma insulin levels. OGTT was performed after fed with high fat emulsion for 14 days. Briefly, after overnight fasting, glucose (2.0 g/kg body wt) was intragastrically administrated and blood glucose levels were measured at 0 (before glucose administration), 0.5 h, 1 h, and 2 h, respectively. The plasma samples were separated and stored at −80°C for fasting insulin detection. Plasma insulin levels were assessed using the iodine [^125^I]-insulin radioimmunoassay kit according to the manufacturer's instructions.

HOMA insulin resistance (HOMA-IR) = FIns  (*μ*U × 10^−3^/L) × FG (mmol/L)/22.5. Area under the glucose curve (AUC) = FG/2 + (0.5 h + 1 h + 2 h) glucose.

### 2.4. Thoracic Aortas Ring Preparation and Vascular Function Analysis

After general anesthesia, the rats' thoracic aortas were quickly removed and placed in cold Krebs-Henseleit (K-H) solution (NaCl 118.4 mmol/L, KCl 4.7 mmol/L, KH_2_PO_4_ 1.2 mmol/L, MgSO_4_ 1.2 mmol/L, NaHCO_3_ 25.0 mmol/L, glucose 11.6 mmol/L, and CaCl_2_ 1.9 mmol/L). Excess tissue and adventitia were carefully removed and two 3~4 mm length rings were cut contiguously from the descending aorta. Vascular tension was measured with a force transducer connected to BL420E + Data Acquisition and Analysis System (Chengdu, China). Two stainless-steel stirrups were passed through the lumen of each ring in order to measure the tension. The rings were placed in 10 mL organ chambers containing K-H solution grassed with 95% O_2_ and 5% CO_2_, which was maintained at 37°C. The rings were initially stretched to a tension of 0.2 g, allowed to equilibrate for 30 min, and mechanically increased by 0.2 g per 5 min until a basal tension of 2 g in the bath fluid, which was changed every 30 minutes. After equilibration, the rings were contracted with NE (10^−9^~10^−6^ mol/L) firstly. The rings would be discarded if contractile tension was less than 1 g. Vascular rings' relaxation was induced by ACh (10^−8^~10^−5^ mol/L) The vascular contraction was shown by tension (g), and relaxation was shown by relaxation rate (%).

Relaxation (%) = [tension of NE (10^−6^ mol/L) − tension of ACh (10^−8^~10^−5^ mol/L)]/ [tension of NE (10^−6^ mol/L) − 2.0] × 100% [16].

### 2.5. HE and Immunohistochemistry Analysis

Thoracic aortas were fixed with 4% paraformaldehyde, embedded in paraffin, and cross-sectioned (4 *μ*m). After hot incubation for 3 hours and deparaffinization, (1) parallel sections were subjected to standard HE staining, (2) rabbit polyclonal antibody to vWF at 1 : 250 was incubated overnight at 4°C, and then (3) Polink-2 Plus Polymer HRP Detection System for rabbit primary antibody and DAB Detection System were used for the immunohistochemistry analysis of vWF expression. The histological changes of aortas walls were observed under light microscope.

### 2.6. Scanning Electron Microscopic Observation

For electron microscopic observation, thoracic aortas were cut open longitudinally, rinsed with ice-cold 0.1 mol/L phosphate buffer (pH 7.2), and fixed with 3% glutaraldehyde for 2 h After being washed with 0.1 mol/L phosphate buffer for 3 times, the aortas were fixed with osmium tetroxide and dehydrated in ethanol. After critical point dried and sputter-coated with gold, samples were examined and images were acquired using a JEOL JSM-6360LV scanning electron microscope (JEOL, Japan).

### 2.7. Cell Culture and Treatment

This investigation has been conducted according to the principles expressed in the Declaration of Helsinki. Umbilical cords were obtained from healthy births at Beijing Friendship Hospital, Capital Medical University with written informed consent, in accordance with the guidelines of research ethics committee of Capital Medical University, and had been specifically approved by this committee. Human umbilical cords endothelial cells (HUVECs) were isolated from fresh human umbilical cords as previously described [25]. The endothelial cells were identified by the presence of eNOS and VE-cadherin antigens and a typical “cobble-stone” appearance. Endothelial cells of the third to fifth passages in the actively condition were used for experiments.

To assess the effectiveness of high glucose induced injury in HUVECs, cells were incubated with different concentrations of exogenous glucose (0, 5, 10, 15, 20, and 25 mmol/L, except for the 5 mmol/L glucose in DMEM itself) for 48 hours in the prepresence or absence of different concentrations of exendin-4 (the active ingredient of exenatide) and then underwent cell viability and apoptosis assays.

### 2.8. Cell Viability Assay

For cell viability assay, HUVECs were seeded on 96-well plates and exposed to various treatments. In the exendin-4 prepresence group, HUVECs were firstly treated with exendin-4 for 30 minutes before being incubated with glucose for 48 hours. Cells were treated with 100 *μ*L MTT solution (the final concentration was 0.5 mg/mL) at 37°C for additional 4 hours. The formazan crystals were solubilized with 100 *μ*L DMSO and then the absorbance was measured at wavelength of 540 nm using a microplate reader (Bio-Tek Instruments, Winooski, VT, USA).

### 2.9. Cell Apoptosis Assays

The apoptosis of HUVECs was determined by a combination of histochemical staining (terminal deoxynucleotidyl transferase-mediated dUTP nick-end labelling staining (TUNEL)) and caspase-3 activity detection.

Cells were fixed in 4% paraformaldehyde and washed twice. The cells were firstly incubated with 20 *μ*g/mL proteinase K for 15 minutes, rinsed with phosphate buffered saline (PBS), then incubated with 3% H_2_O_2_ and methanol to block the endogenous peroxidase activity, and then rinsed with PBS. The slides were stained with an in situ cell death detection POD kit in accordance with the manufacturer's instructions. All slides were counterstained with hematoxylin. As a negative control, the terminal transferase was omitted. Immunofluorescence staining was performed using the immunofluorescence marker included in the kit. The TUNEL positive cells were found before DAB coupling. After this pilot evaluation, all the slides were stained by DAB coupling. Photos were captured under a light microscope and positive cells were counted using Image J software.

The activity of caspase-3, a final common pathway in caspase-dependent apoptosis, was determined by chemiluminescence as we described previously [26]. The substrate Ac-DEVD-AFC was used to determine caspase-3 activity according to the manufacturer's instructions. Briefly, cell tissues were homogenized in ice-cold lysis buffer and centrifuged at 12,000 rpm for 10 min at 4°C; 50 *μ*L of supernatant was then incubated with buffer containing 10 mM dithiothreitol and 5 *μ*L Ac-DEVD-AFC (the final concentration was 200 *μ*M) at 37°C for 1.5 h. Activity of caspase-3 was determined using a spectrophotometer at 405 nm (Molecular Devices, Sunnyvale, CA), and the results were expressed as—fold of the vehicle group.

### 2.10. Measurement of Nitrotyrosine Content

The nitrotyrosine content in rat plasma and HUVECs homogenates was detected with an ELISA kit, as described previously [27, 28]. The results were presented as nmol of nitrotyrosine/litre in the plasma and nmol of nitrotyrosine/g of protein in the cell homogenates.

### 2.11. Western Blotting Analysis of Inducible Nitric Oxide Synthase (iNOS) in HUVECs

HUVECs were lysed in RIPA protein lysis buffer. A total of 50 *μ*g protein was loaded to 8% sodium dodecyl sulfate-polyacrylamide gel. Proteins were transferred to PVDF Sequi-Blot membranes (BioRad). Nonspecific binding sites were blocked for 2 h with 5% nonfat dried milk in TBS (20 mM Tris-Base, 0.5 M NaCl, pH7.4). The membranes were incubated overnight at 4°C with mouse anti-NOS2 (iNOS) monoclonal antibody (1 : 500) and the loading control rabbit anti-*β*-actin monoclonal antibody (1 : 2500). After washing, the membranes were incubated for 2 h at room temperature with secondary antibodies. The antigens were detected by the luminescence method using SuperSignal West Pico Chemiluminescent Substrate kit. After immunoblotting, the film was scanned and intensity of immunoblot bands was detected with Quantitative-One software (Gel Doc 2000 Imaging System, Bio-Rad, Hercules, CA, USA).

### 2.12. Measurement of MDA Content in HUVECs

MDA is a stable metabolite of the ROS-mediated lipid peroxidation cascade, often used as index of oxidative stress. HUVECs homogenates were centrifuged at 4°C, 12000 rpm, for 10 min. The total protein concentration was determined by the Bradford method. The MDA content was measured using a commercial kit as described previously [29]. The MDA content of the homogenates was determined spectrophotometrically and was expressed as nmol/g of protein.

### 2.13. Statistical Analysis

Statistical analysis was performed with SPSS 13.0. Repeated measures analysis of variance (ANOVA) followed by Bonferroni post hoc test was used to assess the statistical differences of vascular responses to NE and ACh among groups. One-way ANOVA followed by Student-Newman-Keuls post hoc test was used to analyze the statistical differences of other data. Statistical significance was considered at the *P* < 0.05 level.

## 3. Results

### 3.1. The Rats DM Models Had Been Successfully Established and Treatment with Exenatide Decreased Fasting Glucose Levels in DM Rats

**Figure 1:**
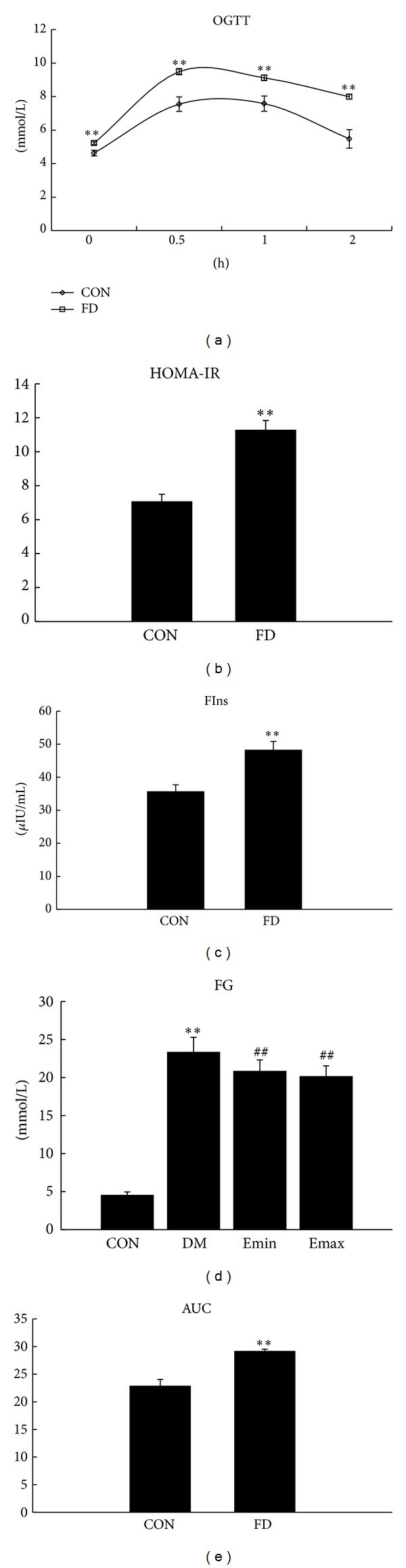
The rats DM models had been successfully established and treatment with exenatide decreased fasting glucose levels in DM rats. (a) The changes of OGTT. High fat diet induced a significant increase in blood glucose levels in every time point. (b) Fasting insulin levels (FIns). High fat diet induced a significant increase in fasting insulin levels. (c) The changes of AUC. High fat diet induced a significant increase in AUC (AUC = FG/2 + (0.5 h + 1 h + 2 h) glucose). (d) HOMA insulin resistance (HOMA-IR). High fat diet induced a significant increase in HOMA-IR (HOMA-IR = FIns (*μ*U × 10^−3^/L) × FG (mmol/L)/22.5). (e) Fasting glucose (FG) levels. DM induced a significant increase in fasting glucose levels, and treatment with exenatide for 45 days significantly decreased fasting glucose levels. In (a), (b), (c), and (d), CON *n* = 7 and FD *n* = 27. In (e) CON *n* = 7; DM, *E*_min⁡_, and *E*_max⁡_ *n* = 9. **P < 0.01 versus CON; ^##^ *P* < 0.01 versus DM, mean ± SEM.

Insulin resistance refers to the diminished effectiveness of insulin in lowering blood glucose levels, which is the main initial and independent risk factor for type 2 diabetes mellitus [30]. Insulin resistance is usually associated with obesity and can be induced by long-term high fat or/and high glucose diet [31, 32]. In this study, we fed the rats with high fat emulsion in order to induce insulin resistance. After high fat diet for 14 days, there was a significant increase in blood glucose levels at every time point of OGTT (Figure 1(a), *P* < 0.01 versus CON) and fasting insulin levels (Figure 1(b), *P* < 0.01 versus CON). Calculated AUC and HOMA-IR were also increased significantly (Figure 1(c), *P* < 0.01 and Figure 1(d), *P* < 0.01 versus CON, resp.). Furthermore, after injection of low dose of streptozotocin twice, there was a significant increase in fasting glucose levels in fat diet rats comparing with normal rats (Figure 1(e), *P* < 0.01), indicating that the DM models had been successfully made. Treatment with exenatide (either *E*_min⁡_ or *E*_max⁡_) for 45 days effectively attenuated the increase in glucose levels in DM rats (Figure 1(e), both *P* < 0.01 versus DM). There was no significant difference between the two treatment groups.

### 3.2. Treatment with Exenatide Significantly Improved Vascular Endothelium-Dependent Vasodilatation in DM Rats

**Figure 2:**
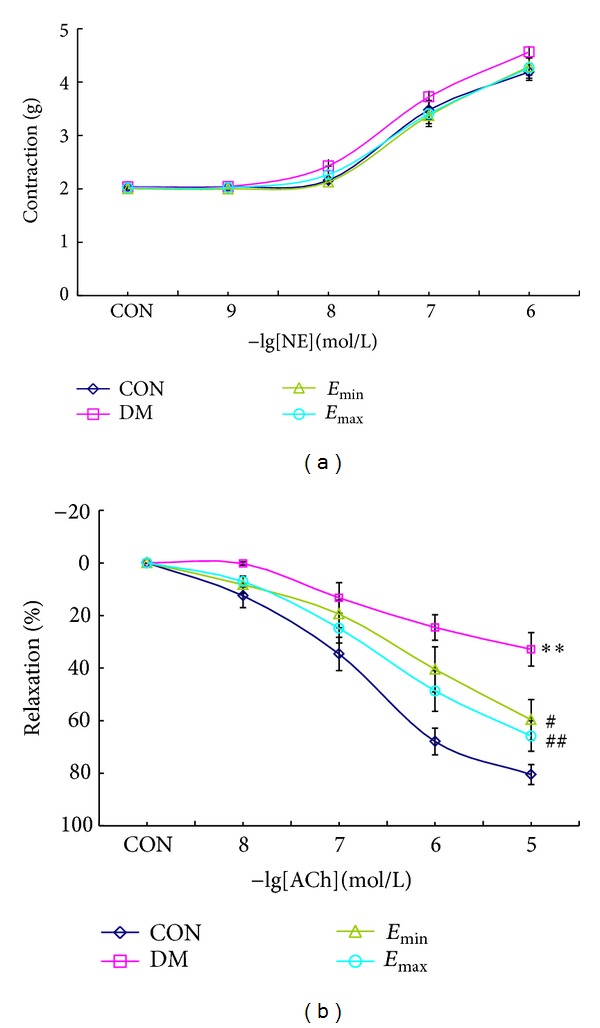
Treatment with exenatide significantly improved endothelium-dependent vasodilatation in DM rats. (a) Responses of thoracic aortas rings to increasing concentration of NE. There was no significant difference in NE elicited contraction among groups. (b) Responses of thoracic aortas rings to increasing concentration of ACh. DM induced a significant decrease in relaxation of ACh, and treatment with exenatide for 45 days significantly improved the relaxation of ACh. CON *n* = 7; DM, *E*_min⁡_, and *E*_max⁡_ *n* = 9. **P < 0.01 versus CON; ^#^ *P* < 0.05, ^##^ *P* < 0.05 versus DM, mean ± SEM.

To elucidate the effect of exenatide on vascular function, we investigated the change of vasoconstriction and vasodilation in different groups of rats. In thoracic aortas rings, NE (10^−9^~10^−6^ mol/L) elicited obvious contraction in a dose-dependent manner, and there was no significant difference in NE elicited contraction among groups (Figure 2(a)), implying that there was no obvious smooth muscle cells damage. ACh can stimulate the endothelial cells to release nitric oxide (NO) and then induce the relaxation of smooth muscle cells. The vascular responses to ACh reflect the function of endothelium. In DM group, the ACh elicited relaxation was significantly weakened compared to CON group (*P* < 0.01), and exenatide treatment significantly attenuated this decline of relaxation (Figure 2(b), *E*_min⁡_ *P* < 0.05 and *E*_max⁡_ *P* < 0.01, resp., versus DM). All these results suggested that exenatide could improve endothelial dysfunction induced by DM.

### 3.3. Treatment with Exenatide Attenuated Thoracic Aortic Histological Changes in DM Rats

**Figure 3:**
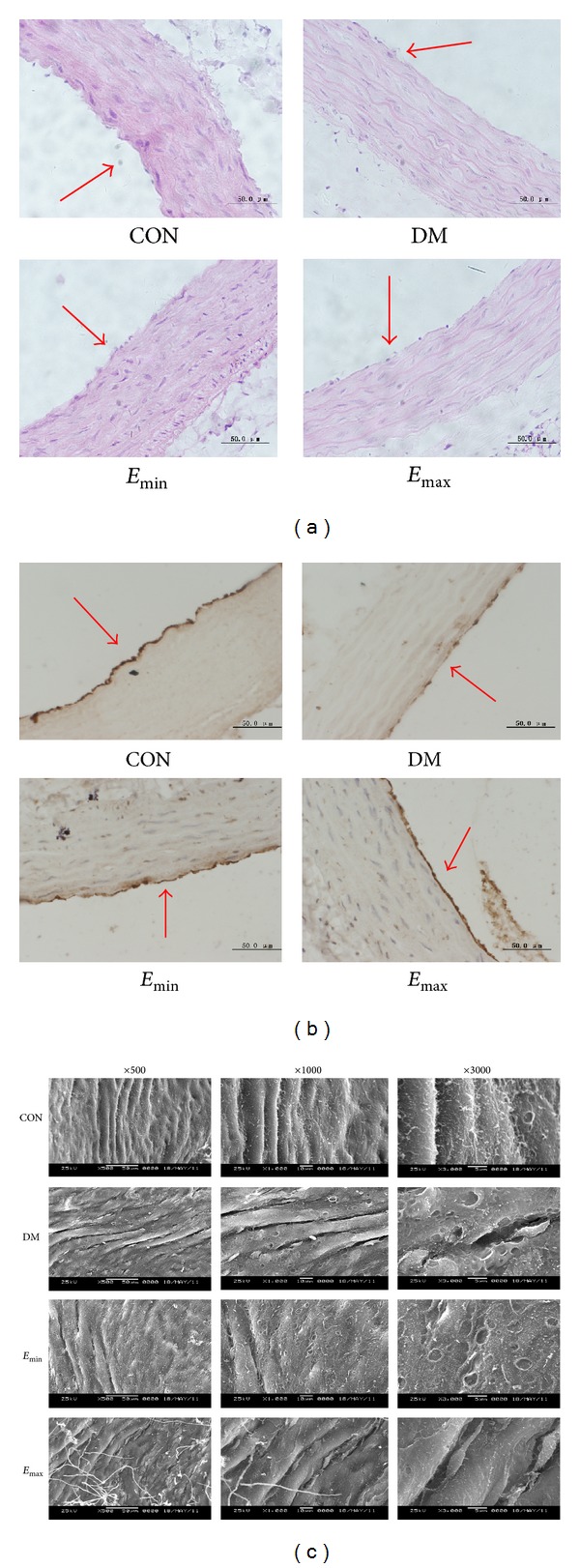
Treatment with exenatide attenuated thoracic aortic histological changes in DM rats. (a) HE staining (×40). (b) vWF immunohistochemistry staining (×40). (c) Scanning electron microscopic observation (×500, ×1000, and ×3000). In DM group, the vascular endothelium and internal elastic membrane were severely damaged. There were less vWF positive particles and the continuity of the vWF positive thoracic aorta wall was interrupted. Endothelial cells were atrophic, cell junctions were damaged, moth-eaten injury was serious in cell membrane, mitochondria were swelling, lamellar cristae disappeared, and matrix was diluted. Treatment with exenatide improved the vascular endothelium in DM rats. Smooth muscle cells were almost normal in all groups.

Next we assessed the effect of exenatide on thoracic aortic histological changes in DM rats. Concomitant with the improvement of endothelial function, the endothelial histological changes in DM rats were also improved. HE staining showed that the vascular tunica intima in DM was severely damaged: the vascular endothelium staining was shallow and the internal elastic membrane was broken (Figure 3(a)). vWF is a specific marker for endothelium; the immunohistochemistry staining showed that less vWF positive particles appeared and the continuity of the vWF positive thoracic aorta wall was interrupted in DM rats, indicating obvious vascular endothelial injury. Treatment with exenatide attenuated the endothelial injury induced by DM. Smooth muscle cells were almost normal in all groups overall (Figure 3(b)). Scanning electron microscopic (SEM) observation also showed that the impairment of endothelium in DM was serious: endothelial cells were atrophic with intercellular space widened, intercellular junctions were partly damaged, and moth-eaten injury was serious on cell membrane. Treatment with exenatide attenuated the vascular endothelium ultrastructural injury too (Figure 3(c)).

### 3.4. Treatment with Exenatide Decreased the Plasma Nitrotyrosine Concentration in DM Rats

**Figure 4:**
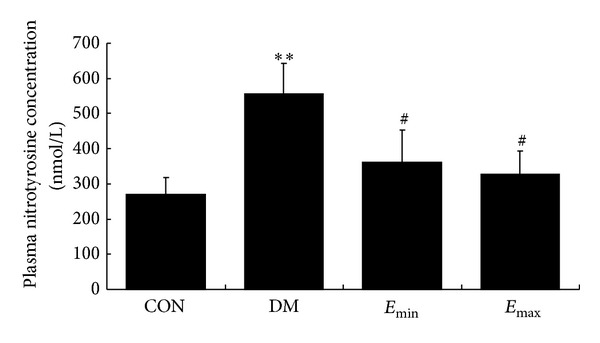
Treatment with exenatide decreased the plasma nitrotyrosine concentration in DM rats. DM induced a significant increase in the plsma nitrotyrosine concentration, and treatment with exenatide for 45 days significantly decreased the plsma nitrotyrosine concentration. CON, *n* = 7; DM, *E*_min⁡_, and *E*_max⁡_ *n* = 9. **P < 0.01 versus CON; ^#^ *P* < 0.05 versus DM, mean ± SEM.

Peroxynitrite (ONOO^−^) can initiate both nitrative and oxidative reactions with proteins, lipids, and DNA. A characteristic reaction of ONOO^−^ is the nitration of protein-bound tyrosine residues to produce nitrotyrosine, and the production of nitrotyrosine has been used extensively as a footprint for ONOO^−^ in vivo. Recent evidence has indicated that the vascular endothelial injury induced by hyperglycemia is associated with the enhanced nitrooxidative stress both in vivo and in vitro [3–6]. To explore the possible mechanisms involved in the endothelium-protective effect of exenatide, we furthermore investigated the effect of exenatide on plasma nitrotyrosine concentration in DM rats. ELISA analysis revealed that the plasma nitrotyrosine concentration in DM rats was significantly increased (*P* < 0.01 versus CON), and treatment with exenatide for 45 days antagonized the increase in plasma nitrotyrosine concentration (*P* < 0.05 versus DM). There was no significant difference between the two treatment groups (Figure 4). These results primarily implied that the alteration of nitrative stress in rats was parallel with the vascular endothelial injury among groups.

### 3.5. Exendin-4 Attenuated Highglucose Induced Endothelial Injury In Vitro

**Figure 5:**
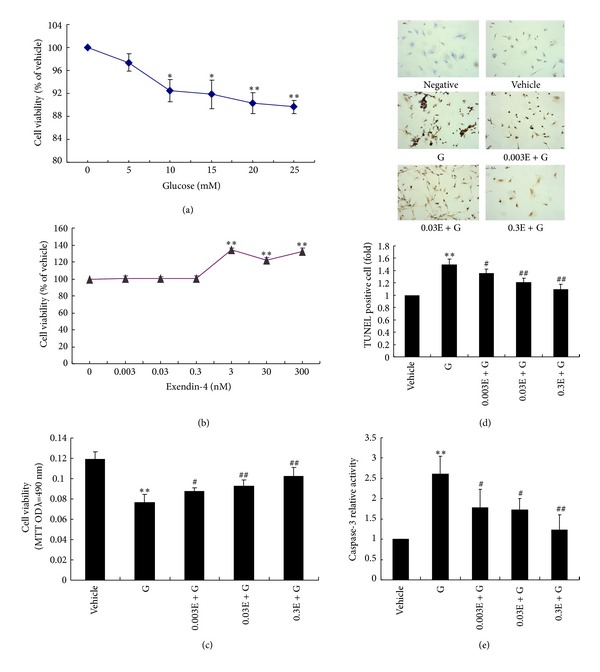
Exendin-4 attenuated high glucose induced endothelial injury in vitro. (a) Highglucose induced a decreased cell viability of HUVECs. (b) Effect of different dose of exendin-4 on cell viability of HUVECs. (c) Exendin-4 pretreatment improved cell viability challenged with highglucose. (d) Exendin-4 pretreatment alleviated the increased TUNEL positive cells induced by highglucose. (e) Exendin-4 pretreatment alleviated the increased caspase-3 activity induced by high glucose. *n* = 5~10. *P < 0.05, **P < 0.01 versus vehicle; ^#^ *P* < 0.05, ^##^ *P* < 0.01 versus G. G: 10 mM high glucose; 0.003E: 0.003 nM exendin-4; 0.03E: 0.03 nM exendin-4; 0.3E: 0.3 nM exendin-4.

Furthermore, we administered high glucose to induce endothelial injury in cultured HUVECs in vitro and assess the effect of exendin-4, the active ingredient of exenatide, on cell apoptosis. HUVECs were identified by cobblestone-like morphology and immunofluorescence staining of eNOS and VE-cadherin (see Figure 1 in Supplementary material available online at http://dx.doi.org/10.1155/2013/843657). High glucose potently diminished the number of viable HUVECs in a dose-dependent manner (Figure 5(a)). We found that exendin-4 alone, at lower concentration (0.003, 0.03, and 0.3 nmol/L) had no obvious influence on HUVECs, while higher concentration (3.0, 30, and 300 nmol/L) exendin-4 promoted HUVECs proliferation (Figure 5(b)). In consideration of the mean peak concentration of exendin-4 (211 pg/mL, i.e., 0.05 nmol/L) in vivo in clinic, and also to avoid the compound proliferative effect of high concentration of exendin-4 on HUVECs, we selected the lower concentration (0.003, 0.03 and 0.3 nmol/L) for stimulating cells in later study. Interestingly, the mean peak concentration of exendin-4 (211 pg/mL, i.e., 0.05 nmol/L) in vivo in clinic is also in this concentration range. Our results showed that exendin-4 pretreatment significantly improved the decreased cell viability (Figure 5(c)) and alleviated the increased TUNEL positive cells (Figure 5(d)) and caspase-3 activity (Figure 5(e)) induced by high glucose. These results showed that exendin-4 pretreatment might protect endothelial cells through attenuating cell apoptosis.

### 3.6. Exendin-4 Attenuated Highglucose Induced Endothelial Nitrooxidative Stress In Vitro

**Figure 6:**
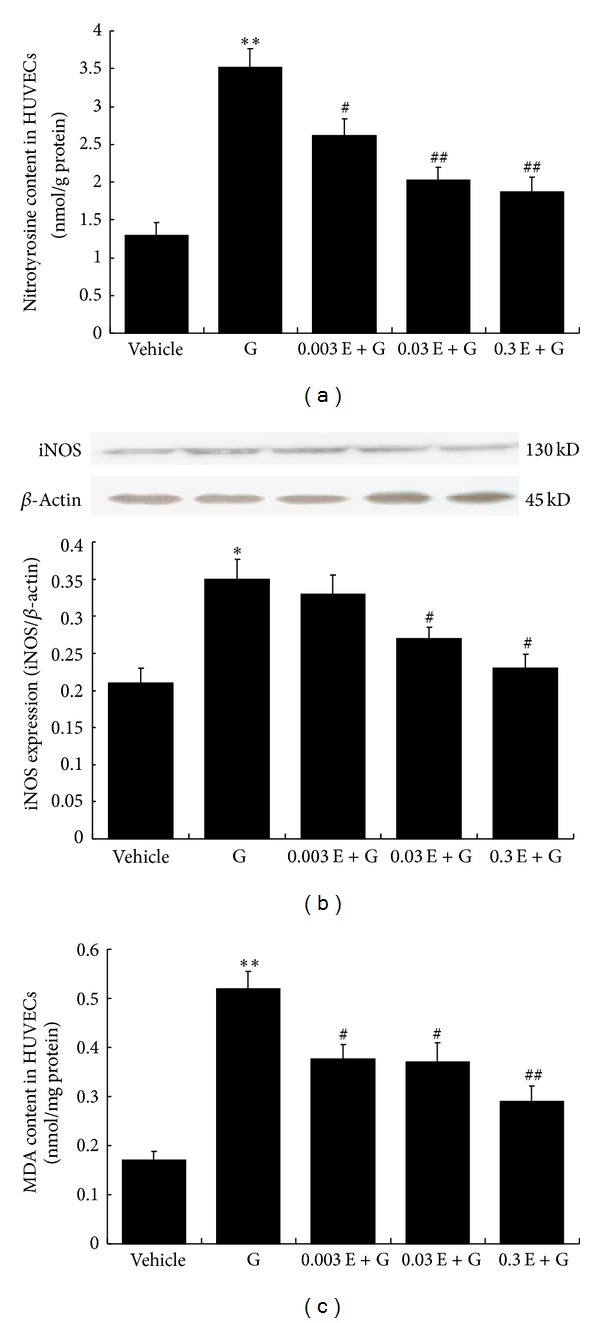
Exendin-4 attenuated highglucose induced endothelial nitrooxidative stress in vitro. (a) Nitrotyrosine content in HUVECs. *n* = 6. (b) MDA content in HUVECs. *n* = 6. (c) iNOS expression in HUVECs. *n* = 4. *P < 0.05, **P < 0.01 versus vehicle; ^#^ *P* < 0.05, ^##^ *P* < 0.01 versus G, mean ± SEM. G: 10 mM high glucose; 0.003E: 0.003 nM exendin-4; 0.03E: 0.03 nM exendin-4; 0.3E: 0.3 nM exendin-4.

To further explore the role of nitrooxidative stress in endothelium-protective action of exenatide, we also detected the nitrotyrosine, MDA content and iNOS expression in different group of HUVECs. When superoxide anion (^∙^O_2_^−^) combines with nitric oxide (NO) at a diffusion-limited rate, it becomes a highly reactive species, peroxynitrite (ONOO^−^). MDA is a stable metabolite of the ROS-mediated lipid peroxidation cascade, often used as index of oxidative stress. The inducible NO synthase (iNOS) is the enzyme catalyzed NO production within pathophysiological conditions, and the elevated iNOS expression usually means the overproduction of NO. Our results showed that high glucose significantly increased the nitrotyrosine, MDA content, and iNOS expression; meanwhile exendin-4 pretreatment significantly attenuated these changes (Figure 6), implying that the endothelium-protective effect of exenatide might be related to the inhibition of nitrooxidative stress.

## 4. Discussion

The main contribution made in the present study is that the alleviation of hyperglycemia induced vascular endothelial injury by exenatide might be related to the inhibition of nitrooxidative stress. Insulin resistance is the main initial and independent risk factor for type 2 diabetes mellitus [30], which is usually associated with obesity, and can be induced by long-term high fat or/and high glucose diet [31, 32]. In this study, we fed the rats with high fat emulsion in order to induce insulin resistance. After high fat diet for 14 days, there was a significant increase in blood glucose levels at every time point of OGTT, AUC, fasting insulin levels, and HOMA-IR, implying that these rats had developed obvious insulin resistance. After that, the rats were injected with low dose of streptozotocin to destruct the pancreatic *β* cells. The fasting glucose levels were increased 72 hours later, which indicated that the DM models had been successfully made. Although this kind of experimental DM models has been reported previously [24], it would be superior if we use a spontaneously established rat model of T2DM, such as the Zucker diabetic fatty (ZDF) rat, a good model for cardiovascular complications of DM. This is one of the limitations of our study.

Exenatide is an incretin mimetic agent that mimics the enhancement of glucose-dependent insulin secretion. It is indicated as adjunctive therapy to improve blood glucose control in patients with T2DM. Our results showed that treatment with exenatide, either low (312.5 × 10^−3^ *μ*g) or high dose (625 × 10^−3^ *μ*g) for 45 days, could significantly reduce fasting blood glucose levels in DM rats. We noticed that the reduction percentage of fasting blood glucose levels was rather less; this may be due to the relatively severe pancreatic injury or without associated drug administration like clinic.

Previous studies have shown that in addition to coordinated effects on glucose metabolism [20, 21] exenatide exerts some similar endothelium-protective action with GLP-1 [22, 23, 33, 34]. In accordance with other reports, we also observed the protective effect of exenatide on endothelium both in vivo and in vitro. Our results showed that treatment with exenatide improved both endothelial functional and histological changes in DM rats. Furthermore, in the highglucose induced HUVECs injury models, our study showed that exendin-4, the active ingredient of exenatide, pretreatment improved cell viability through attenuating cell apoptosis. These results imply that exenatide can protect vascular endothelial cells in hyperglycemia directly. However, the underlying mechanisms are not fully understood.

Hyperglycemia can increase the production of superoxide anion (^∙^O_2_^−^). Although (^∙^O_2_^−^) itself is chemically inert, when it combines with nitric oxide (NO) at a diffusion-limited rate, it becomes a highly reactive species, peroxynitrite (ONOO^−^). ONOO^−^ can initiate both nitrative and oxidative reactions with proteins, lipids, and DNA. A characteristic reaction of ONOO^−^ is the nitration of protein-bound tyrosine residues to produce nitrotyrosine, and the production of nitrotyrosine has been used extensively as a footprint for ONOO^−^ in vivo. Recent evidence has indicated the endothelial injury induced by hyperglycemia is associated with the enhanced nitrooxidative stress both in vivo and in vitro [3–6]. There is report that another GLP-1 analogue sitagliptin, a kind of dipeptidyl peptidase-4 (DPP-4) inhibitor, caused a substantial reduction of nitrative stress within the brain in Alzheimer's prone mice [35]. Is it possible that the endothelium-protective action of exenatide is also related to the reduction of nitrooxidative stress?

In the present study, we found that the plasma nitrotyrosine concentration in DM rats was significantly increased, and treatment with exenatide for 45 days antagonized the elevation of plasma nitrotyrosine concentration. These results implied that the alteration of nitrative reaction in rats was parallel with the vascular endothelial injury among groups. Furthermore, to explore the role of nitrooxidative stress in endothelium-protective action of exenatide, we also detected the nitrotyrosine, MDA content, and iNOS expression in different group of HUVECs. MDA is a stable metabolite of the ROS-mediated lipid peroxidation cascade, often used as index of oxidative stress. High production of (^∙^O_2_^−^) can induce the elevated MDA content. iNOS is the enzyme catalyzed NO production within pathophysiological condition, and the elevated iNOS expression means the high production of NO. Our results showed that high glucose significantly increased the nitrotyrosine, MDA content, and iNOS expression; meanwhile exendin-4 pretreatment significantly attenuated these changes, implying that the endothelium-protective effect of exenatide might be related to the inhibition of nitrooxidative stress. It is our limitation that there is lack of data of NO content in the endothelium because of some technical reasons. Although there is no obvious influence on the final conclusion of this study, we would try to apply superior method to confirm the present finding in the future.

In summary, this study demonstrates that treatment with exenatide can alleviate the vascular endothelial injury, as well as attenuating the ONOO^−^ induced nitrooxidative stress in hyperglycemia, implying that the endothelium-protective effect of exenatide might be related to the reduction of nitrooxidative stress. Although the detailed mechanisms responsible for this effect still remain to be explored, these new insights gained from the current study may shed a novel light on better mechanistic understanding of exenatide.

## Supplementary Material

Characterization of primarily cultured human umbilical vein endothelial cells (HUVECs). (a) After 5 days of feeding, cells isolated from human umbilical cord vein showed a typical, cobblestone-like morphology of endothelial cells. (b) The markers of endothelial cells (eNOS and VE-cadherin) were positive with immunofluorescence staining (×100).
