# Supplementary figures and images for: Alleviation of Hyperglycemia Induced Vascular Endothelial Injury by Exenatide Might Be Related to the Reduction of Nitrooxidative Stress
Source: Biomed Res Int. 2013 Nov 26;2013:843657. doi: 10.1155/2013/843657 (PMC3858999; doi:10.1155/2013/843657)

A

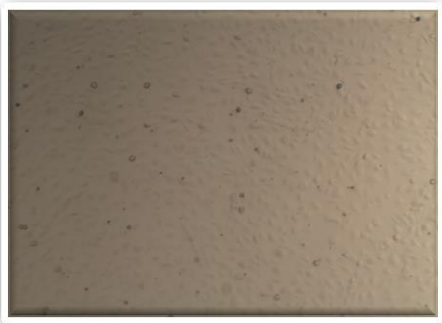

× 40

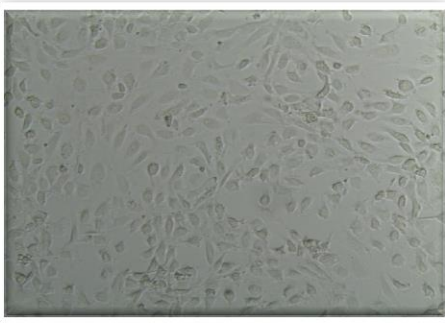

× 100

B

eNOS

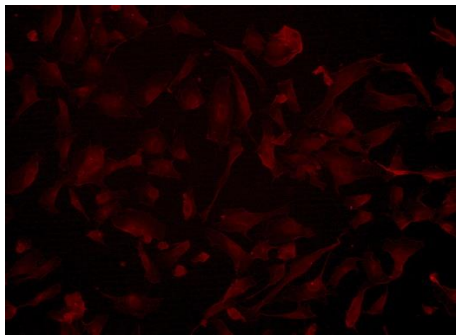

DAPI

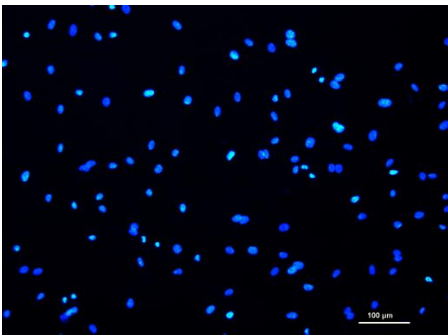

Merge

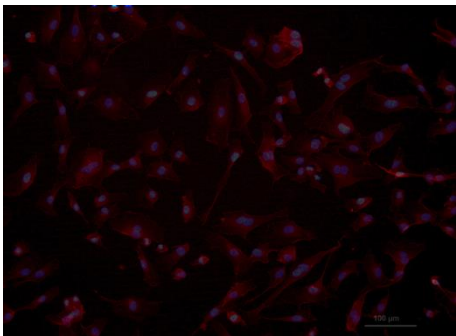

VE-cadherin

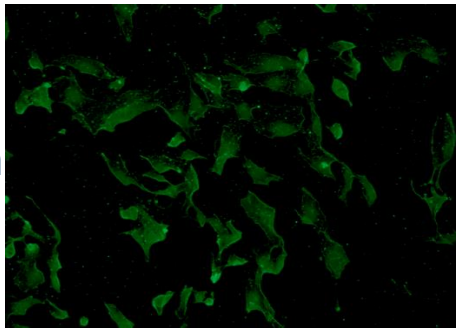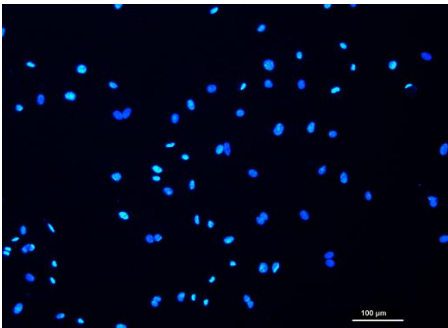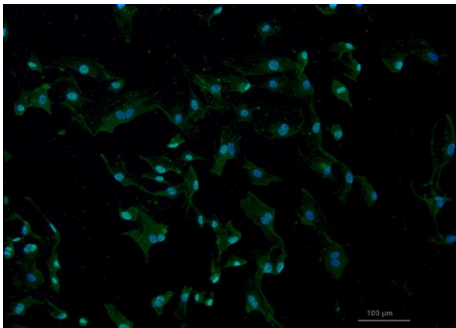

× 100

Supplement: Supplementary file 1 — Characterization of primarily cultured human umbilical vein endothelial cells (HUVECs). (a) After 5 days of feeding, cells isolated from human umbilical cord vein showed a typical, cobblestone-like morphology of endothelial cells. (b) The markers of endothelial cells (eNOS and VE-cadherin) were positive with immunofluorescence staining (×100). [file 843657.f1.pdf]
